# Target variability and stability of neuroimaging-guided transcranial magnetic stimulation of the amygdala circuitry for posttraumatic stress disorder

**DOI:** 10.21203/rs.3.rs-8321466/v2

**Published:** 2026-01-26

**Authors:** Sanne J.H. van Rooij, Cecilia A. Hinojosa, Patlapa Sompolpong, Malin Au, Lois Teye-Botchway, Timothy D. Ely, Gregory Job, Sean T. Minton, Ryan Langhinrichsen-Rohling, Patricio Riva-Posse, Joshua Lukemire, Kerry J. Ressler, Nadine J. Kaslow, Sheila A.M. Rauch, Tanja Jovanovic, Paul E. Holtzheimer, Vince D. Calhoun, William M. McDonald, Joan A. Camprodon

**Affiliations:** 1 Emory University School of Medicine, Department of Psychiatry and Behavioral Sciences; 2 University of New Mexico, Department of Psychology; 3 Emory University Laney Graduate School, Graduate Division of Biological and Biomedical Sciences; 4 Amsterdam University Medical Center, Department of Psychiatry; 5 Emory University Rollins School of Public Health, Department of Biostatistics and Bioinformatics; 6 Harvard Medical School, Department of Psychiatry; 7 McLean Hospital, Division of Depression and Anxiety Disorders; 8 Joseph Maxwell Cleland Atlanta VA Healthcare System; 9 Wayne State University Department of Psychiatry and Behavioral Neurosciences; 10 National Center for PTSD; 11 Dartmouth Geisel School of Medicine, Departments of Psychiatry and Surgery; 12 Tri-institutional Center for Translational Research in Neuroimaging and Data Science, Georgia State, Georgia Tech; 13 Mass General Brigham, Division of Neuropsychiatry and Interventional Psychiatry

**Keywords:** transcranial magnetic stimulation (TMS), neuronavigation, resting state functional connectivity (rs-FC), functional magnetic resonance imaging (fMRI), precision psychiatry, threat neurocircuitry

## Abstract

**Background:**

Transcranial magnetic stimulation (TMS) is a non-invasive neuromodulation therapy that is applied across psychiatric conditions to modulate specific neural circuits and improve clinical symptoms. While functional magnetic resonance imaging (fMRI)-guided personalized TMS targets are increasingly used, there are critical unresolved methodological, neurobiological, and clinical questions. Addressing topographic variability, stability, and associations with clinical outcomes is essential for advancing clinical development and scalable precision neuromodulation.

**Methods:**

A precision neurocircuitry-based fMRI-guided TMS approach was developed to treat disorders of the amygdala. In a randomized clinical trial for posttraumatic stress disorder (PTSD; n=50), topographic variability and stability of patient-specific right dorsolateral prefrontal cortex (rDLPFC) targets with the strongest functional connectivity to the right amygdala were analyzed.

**Results:**

There was significant target variability between participants and between targeting methods, but target stability was observed after engaging the amygdala circuitry with behavioral threat-related tasks. Target topography did not change after 20 sessions of sham TMS. However, after active TMS (1Hz, 36,000 pulses) target topography was significantly different. A larger change in the medial-anterior direction correlated with greater PTSD symptom improvement.

**Conclusions:**

Target variability and stability for fMRI-guided TMS of the amygdala circuitry is demonstrated, supporting the use of patient-specific targeting strategies for TMS. A clinical change in PTSD symptoms was associated with greater change in target topography, which suggests neuroplastic adaptations in the targeted networks and a possible treatment-dependent shift towards more medial prefrontal control over amygdala regulation. These findings are important for fMRI-guided precision neuromodulation therapy development, particularly for the amygdala circuitry.

## 1. Introduction

Increasing knowledge of the circuit-level pathophysiology of psychiatric disorders has catalyzed interest in neuromodulation as a therapeutic modality. Indications of clinical application of transcranial magnetic stimulation (TMS) have been expanded given its capacity to modulate specific neural circuits across brain disorders, including posttraumatic stress disorder (PTSD) (1–4). Despite the growing interest and use of personalized TMS targets (5), there are key unanswered questions regarding the topographic variability and stability of personalized targets that are important to consider for the clinical development and scalable implementation of precision neuromodulation. In this randomized controlled study, we developed functional magnetic resonance imaging (fMRI)-guided identification of patient-specific cortical targets for TMS treatment of disorders with aberrant amygdala circuitry. We investigated target variability and stability between individuals and fMRI targeting methods, and across state-dependent dynamics of engaging the targeted circuit with behavioral tasks. Additionally, we investigated TMS target topography after modulating the circuit with 20 sessions of TMS compared to sham.

There have been significant advances in developing precision neuromodulation strategies that identify patient-specific targets for TMS. These innovations align with the increasing emphasis on precision medicine developments in psychiatry and are motivated by the strong evidence for inter-individual heterogeneity in the topography of functional circuits (6). The first use of individualized TMS targeting was based on structural MRI scans, representing an important scientific progression from the use of scalp-based landmarks and promoting reliability across stimulation sessions. However, the correlation between the sulcal and gyral anatomy captured by structural MRI scans and the functional neuroanatomy of distributed networks of cognition and emotion is low. Defining individualized targets based on functional architecture presents a more promising strategy. Efforts in this area have led to the first FDA-cleared treatment in psychiatry to be informed by an imaging biomarker, i.e., the SNT protocol that uses fMRI-guided TMS for the treatment of depression (7,8).

The maladaptive dynamics in the amygdala – a deep mid-temporal brain structure central to threat processing – along with its integrated circuitry, have been identified as central to the pathophysiology of anxiety and negative valence disorders, including PTSD (9). Interdisciplinary research has consistently shown that dysfunction in fear learning and threat detection circuits predicts both the onset and persistence of PTSD (10–15). Therefore, modulating these circuits with TMS could be a promising treatment approach (1,16,17). Neuromodulation therapies, such as TMS, have both local effects on the directly stimulated cortical region (18) and distal network effects to interconnected cortical and subcortical areas (19,20). Indeed, the amygdala can be engaged through stimulation of the area in the prefrontal cortex (PFC) that is positively connected to the amygdala (21). The right dorsolateral prefrontal cortex (rDLPFC) provides a feasible point of access for amygdala circuit neuromodulation, and low-frequency TMS to the DLPFC is effective for reducing PTSD symptoms (1,2). Moreover, our clinical trial showed a reduction in amygdala threat reactivity and PTSD symptoms using this approach. Importantly, the specific location of the PFC subregion that optimally connects with the amygdala varies across individuals. Therefore, identifying individualized DLPFC targets for effective engagement of pathological amygdala dynamics, as in the case of PTSD therapies, is of critical importance.

There is great excitement for individualized fMRI-based targeting and its potential to increase TMS efficacy by delivering more targeted stimulation. While evidence is growing pointing to the added therapeutic benefit of image-guided precision TMS (22,23), there is also debate about the need for individualized targeting because scalp-based accelerated protocols have also shown large effect sizes (24). Moreover, there are important practical considerations: the requirement of a functional MRI scan and the need to process and evaluate the target selection is costly, time-intensive, and a potential obstacle in the scalable implementation of this methodology without evidence for its added efficacy. Additionally, major technical and neurobiological questions associated with the inherent variability of the fMRI blood oxygenation-level dependent (BOLD) signal need to be clarified to successfully implement individualized precision targeting.

First, the variability between individuals needs to be captured for each specific target and analytical strategy to justify its application instead of using the scalp-based or Beam F3(F4) target. Second, there is no clarity regarding the method for defining the target: some studies use negative or anti-correlation between the subcortical region and the cortical target, whereas others use positive FC. The topographic implications and variability of the targets across analytical methodologies needs to be better understood. Moreover, it is critical to determine the stability of the targets over time, particularly in the context of state-dependent dynamics such as changes in mental states. Finally, when the circuit is modulated with active TMS, it is essential to investigate the effect on TMS target location and evaluate its clinical implications.

Here we present a novel precision TMS treatment strategy for PTSD informed by extensive cross-modality research pointing to the (right) amygdala as the driver of PTSD and therapy non-response (14,16). Specifically, we defined every individual’s TMS target within the rDLPFC using resting state functional connectivity (rs-FC). We identified the cortical cluster of voxels with the strongest positive correlation in rs-FC with the right amygdala. For this methodology to be relevant and useful in clinical practice, we hypothesized that 1) TMS target location would be *variable* between subjects, such that individualization of the target results in meaningfully different treatment plans; 2) TMS target location would be *variable* between methods, such that targets defined using the positive or negative rs-FC peak would be significantly different from each other, thus highlighting the importance of choosing the appropriate analytical strategy; and 3) TMS target location would be *stable* within subjects before and after modulating disease-relevant mental states by engaging the amygdala circuitry with a threat behavioral task.

Participants with PTSD symptoms were randomized to twenty sessions of 1Hz TMS (1800 pulses) or sham in a randomized clinical trial using these targets. A follow-up fMRI scan was collected after completing TMS. We tested two additional hypotheses comparing pre- and post-TMS targets: 4) TMS target location would be *stable* within subjects over time, i.e., during a 2-week course of sham TMS; and 5) TMS target location would *change* following a course of 20 sessions (36,000 pulses) of active 1 Hz TMS to the personalized rDLPFC target.

## 2. Methods

### 2.1 Overview study and participants

**Figure 1.**
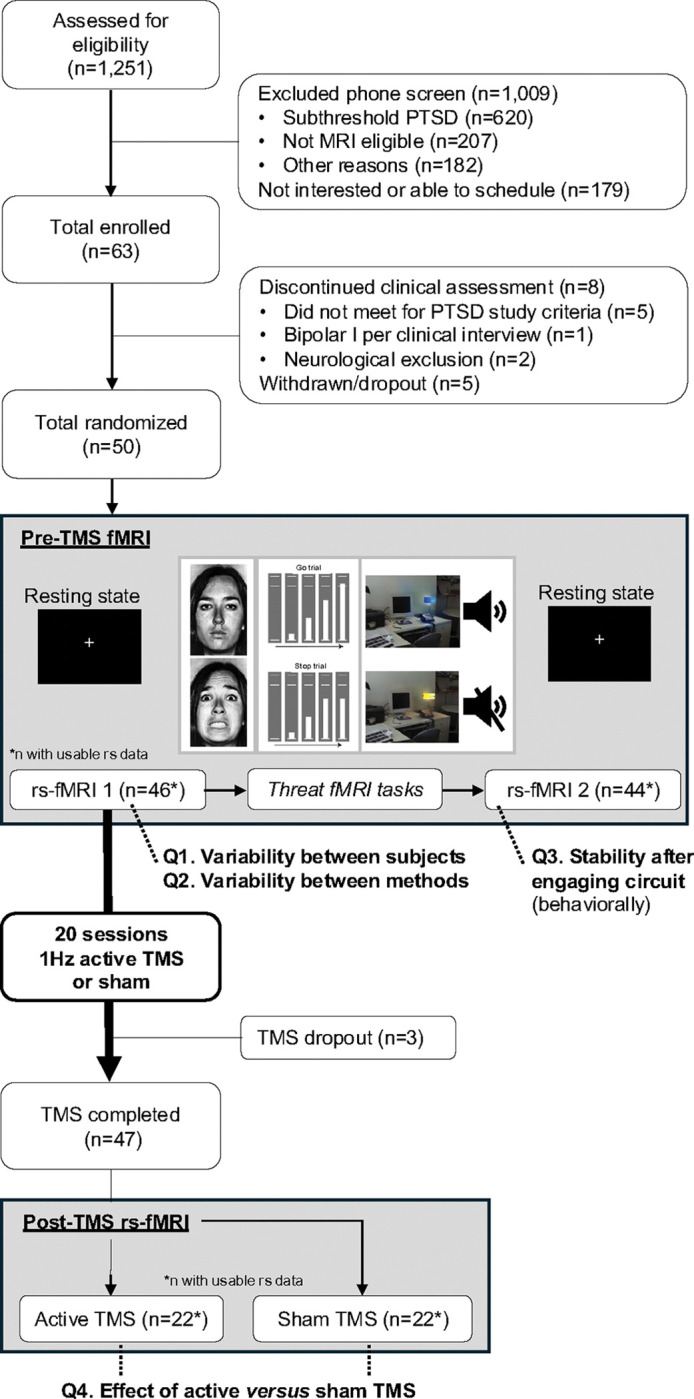
CONSORT diagram and study overview A CONSORT diagram and schematic overview of the clinical trial (https://clinicaltrials.gov/study/NCT04563078). A total of 1,251 participants were assessed for eligibility, 63 were randomized and 50 participants were randomized to two weeks (20 sessions) of 1Hz active or sham TMS. Prior to the first TMS session, an functional magnetic resonance imaging (fMRI) scan was collected. On the scan day, two resting state fMRI scans were collected, one before and after functional engagement of the threat neurocircuitry using fMRI paradigms. The fMRI paradigms included social threat processing (left), contextual response inhibition using the Stop Signal Anticipation Task (middle) and fear conditioning (right). The social threat processing task is based on our previous work with trauma-exposed civilians (11,12,37). Participants passively viewed blocks of fearful and neutral face stimuli, with emotion condition randomly interleaved. Contextual response inhibition was measured with the Stop Signal Anticipation Task (SSAT) following our prior work with trauma-exposed civilians(38). In this task, participants are instructed to stop a moving bar by pressing a button but withhold their response in instances when the bar stops on its own. Contextual cues indicate the chance that the bar stops on its own, allowing for a measure of contextual inhibition. Third, participants completed a *Fear Conditioning* task, also previously used in our research with trauma-exposed civilians.(39) This paradigm was used to measure fear learning by pairing an aversive sound with a yellow or blue lamp. The TMS target used for the clinical trial was defined based on the first resting state scan using positive resting state functional connectivity. Usable targets were available for n=46 and used to measure varability between subjects (Question, Q1). For our analyses of variability between methods (Q2), the anti-correlated or negative target was calculated (n=46). To measure stability of the target (Q3), the target was again defined after engagement of the threat neurocircuitry (n=44 with usable data). After completing 20 session of active or sham TMS (n=47), another fMRI scan was collected. The target was again defined and compared with the pre-TMS target to analyze the effect of active versus sham TMS (Q4) on target topography (n=22, active; n=19, sham).

A randomized double-blind TMS clinical trial for PTSD (https://clinicaltrials.gov/study/NCT04563078) was conducted between February 2021 and March 2025 at Emory University. A total of 1,251 adults (18–65) were screened for eligibility (details in ***Supplemental Materials S1*)**. After the initial phone screen, n=63 (n=36 self-referral) adults with PTSD symptoms were enrolled (Figure 1; CONSORT diagram). The clinician-administered PTSD Scale for DSM-5 (CAPS-5) was used to confirm participants endorsed 3 out of 4 DSM-5 criteria for PTSD (including cluster E, hyperarousal. Fifty adults (Table 1) were randomized to 20 sessions (2 weeks) of active or sham TMS. Forty-seven completed all TMS sessions, and usable fMRI data was available for n=44. The experimental protocols and study procedures were reviewed and approved by the Emory Institutional Review Board (IRB#: STUDY0000038). Written informed consent was obtained from all participants before their inclusion in the study.

### 2.2 MRI scan

The scan protocol is outlined in Figure 1 and scan parameters are listed in Table 2. Two 9.6-minute resting state scans were collected, each consisting of 400 volumes during which participants looked at a white cross on a dark background. In between the two resting state scans, the threat neurocircuitry was engaged with three functional MRI tasks, i.e., social threat processing using fearful faces, contextual response inhibition, and fear conditioning (details in Figure 1). The fMRI tasks were not processed here, and only the resting state scans were used for TMS target identification. Two different scanners were used due to a mid-study scanner upgrade, a 3T Siemens Trio TIM for n=14 and a Prisma Fit (Siemens, Malvern, PA) for n=32. Both scanners used a 32-channel head coil and MRI acquisition parameters were matched (Table 2). To correct for the effect of scanner, we included scanner as a covariate in secondary analysis. Second, to determine consistency across scanners, we separately analyzed MRI scans collected on the Siemens 3T Trio and the Siemens Prisma.

### 2.3 TMS target identification

**Figure 2.**
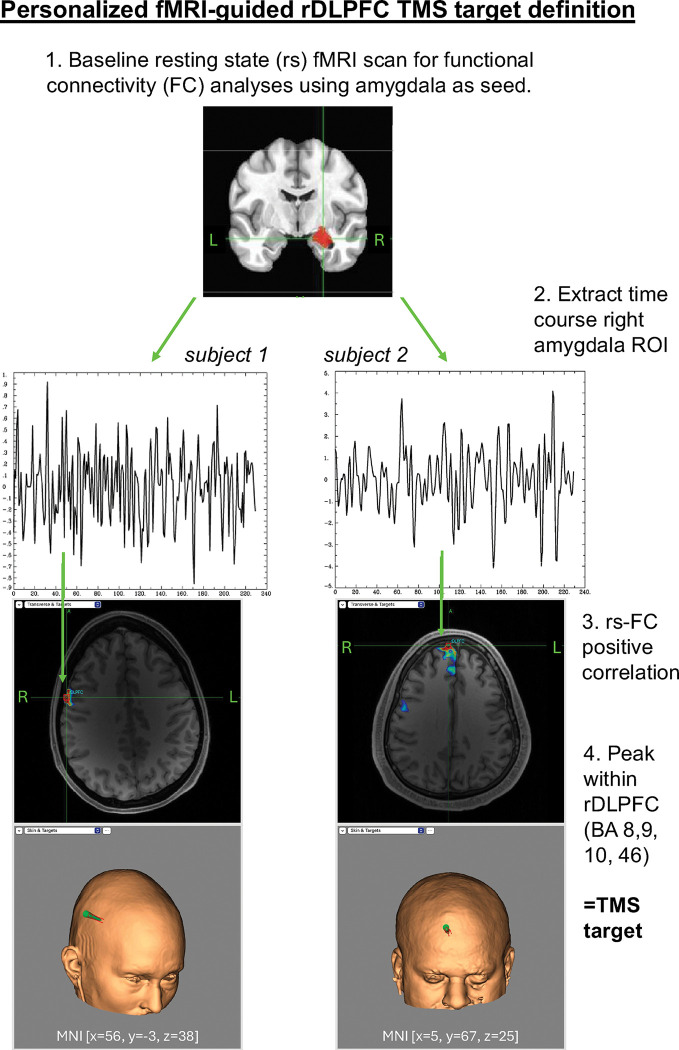
Pipeline for personalized fMRI-guided rDLPFC TMS target definition. Resting state functional MRI was collected prior to transcranial magnetic stimulation (TMS). A correlation map between the extracted timecourse of the right amygdala region of interest (CIT168) and the right dorsolateral prefrontal cortex (rDLPFC) region of interest (BA8, BA9,BA10,BA46; WFU_PickAtlas) was generated and unwarped into native space. Brainsight neuronavigation was used to identify the TMS target. The TMS target in the right dorsolateral prefrontal cortex (rDLPFC) was defined as the positive correlation in functional connectivity to the right amygdala for pre-TMS scan 1. Three criteria were used: 1. Largest positive resting state functional connectivity (rs-FC) peak, 2. Peak within 1.0 cm of the cortex, 3. Location tolerable for 3600 1Hz TMS pulses per day.

Figure 2 shows the pipeline for the fMRI-guided individualized rDLPFC targets, further details in ***Supplemental Materials S2***. TMS stimulation sites were defined by extracting the rs-fMRI timecourse for the right amygdala (identified using the CIT168 atlas (25)) and determining the peak rs-FC within the rDLPFC (defined by integrating BA8, BA9, BA10, BA46; WFU_PickAtlas (26)). Stereotactic neuronavigation (Brainsight) was used to identify the TMS target following three criteria: 1) Largest positive rs-FC peak, 2) Peak within 1.0 cm of the cortex, and 3) Stimulation location tolerable for 3600 1Hz TMS pulses per day for 10 days (as determined by investigator or confirmed with participant). The x, y, and z MNI coordinates were extracted for topographic location.

### 2.4 TMS protocol

The personalized rDLPFC target was used in a clinical trial for PTSD (https://clinicaltrials.gov/study/NCT04563078) using a MagPro X100 with Magoption. The rDLPFC was stimulated twice daily for 10 days with 1Hz TMS (1800 pulses/session, two 30-minute sessions per day, 10-minute break to reduce fatigue) for a total number of 36,000 pulses at 120% motor threshold.

Sham TMS was delivered using the MagPro A/P coil, which mimics the acoustic and tactile sensations of active TMS while attenuating the magnetic field by over 95%. Randomization codes were supplied by MagVenture and managed by an independent researcher not involved in study coordination, data collection, or analysis.

### 2.5 Clinical assessments

To measure PTSD symptoms as an outcome, the PTSD checklist for DSM-5 (PCL-5 (27)) was collected. The PCL-5 was administered by the research assistant after the pre- and post-TMS MRI scans or the closest visit (days since TMS: mean=4.3, SD=3.6, range=0–15). The pre-TMS PCL-5 assessed PTSD symptoms in the past month and the post-TMS PCL-5 in the past week (because the study was conducted in the past month).

### 2.6 Statistical analyses

To determine TMS topography, the x, y, z MNI target coordinates were extracted from Brainsight and exported to SPSS v.29. For all analyses, secondary investigations to consider the different scanners were conducted (***Supplemental Materials S4***). Vectors of change were calculated as

MNI_vector_prepost=SQRT((MNI_X_post–MNI_X_pre)^2+(MNI_Y_post–MNI_Y_pre)^2+(MNI_Y_post–MNI_Y_pre)^2).

1. *Variability between subjects* was investigated by conducting a test of between-subjects effects for the x, y, and z MNI coordinates for positive rs-FC targets in a repeated measures general linear model (GLM).
2. *Variability between methods* was tested by comparing targets defined using positive versus negative (anti-correlation) rs-FC. A 3*2 GLM defining within-subjects variables MNI coordinates for positive and negative rs-FC targets was conducted.
3. *Stability within-subjects after engaging circuit behaviorally* was assessed by comparing positive rs-FC targets before and after engagement of the threat neurocircuitry during the MRI protocol. This was tested with a 3*2 GLM with within-subject variables MNI coordinates for resting state scan 1 and resting state scan 2.
4. *Effect of active versus sham TMS* was investigated by comparing positive rs-FC targets before and after 10 days of active versus sham TMS. A 3*2*2 GLM defining within-subjects variables MNI coordinates for positive rs-FC targets before and after active versus sham TMS was conducted. Post-hoc analyses were conducted if a significant interaction was observed to test the effect of time (sham) or active TMS separately using a 3*2 GLM defining within-subjects variables MNI coordinates for positive rs-FC targets pre- and post-TMS. Exploratory correlation analyses were conducted for symptom improvement (PTSD symptoms pre-TMS minus post-TMS) with change in target location (pre-TMS minus post-TMS). We also conducted correlation analyses to investigate whether the TMS target location (used in the clinical trial) was related to PTSD symptom severity at pre-TMS, post-TMS or symptom change.

## 3. Results

### 3.1 Variability between subjects

**Figure 3.**
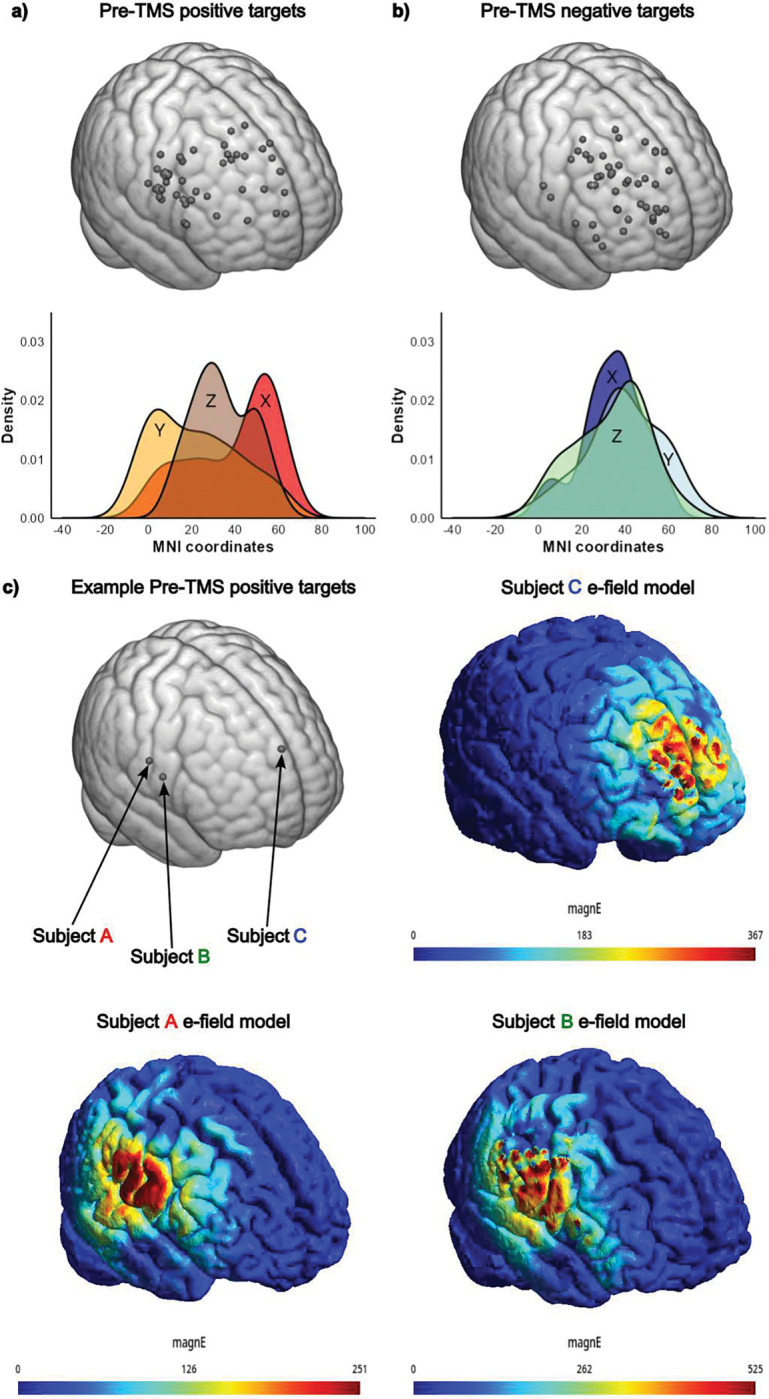
Personalized fMRI-guided rDLPFC TMS target variability between subjects and methods **a.** (Top) Targets in the right dorsolateral prefrontal cortex (rDLPFC) with positive correlation in functional connectivity to the right amygdala for pre-TMS scan 1(positively defined targets; n=46) mapped to a common MNI template. (Bottom) Density plot showing the distribution of the x, y, and z MNI coordinates of positively defined targets. **b.** (Top) Targets in rDLPFC with negative correlation (anti-correlation) in functional connectivity to the right amygdala for the pre-TMS scan 1 (negatively defined targets; n=46) mapped to a common MNI template. (Bottom) Density plot showing the distribution of the x, y, and z MNI coordinates of negatively defined targets. **c.** Example pre-TMS positively defined targets from subjects A, B, and C and their corresponding electric field, or e-field, models simulated using SimNIBS based on each participant’s motor threshold (MT) and maximum stimulator output (MSO), overlayed on their head mesh constructed from T1w structural scans (*Supplemental Materials S2* for details).

Figure 3A displays the fMRI-guided individualized TMS targets used in the TMS clinical trial. The targets showed significant topographic variability between subjects (F_(1,45)_=4,626.43 p<0.001, ηp^2^=0.990). The resulting electrical field models for 3 sample subjects are displayed in Figure 3C.

### 3.2 Variability between methods

Figure 3B shows the targets defined using negative (or anti-correlated) rs-FC. Comparing variability of targeting methods showed significant differences for positive versus negative rs-FC (F_(1,45)_=19.51, p<0.001, ηp^2^=0.302). The average vector of change, calculated across all three x, y, and z directions, was 4.5cm (SD 1.6, range 1.39–8.73).

### 3.3 Stability within-subjects after engaging circuit with behavioral tasks

**Figure 4.**
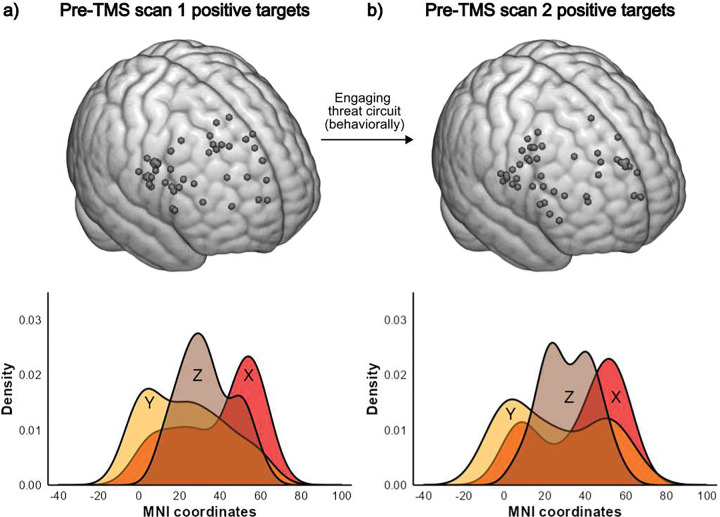
TMS target stability after engaging threat neurocircuit behaviorally **a.** (Top) Targets in the right dorsolateral prefrontal cortex (rDLPFC) with positive correlation in functional connectivity to the right amygdala for pre-TMS scan 1(positively defined targets; n = 44) mapped to a common MNI template. (Bottom) Density plot showing the distribution of the x, y, and z MNI coordinates of pre-TMS positively defined targets using scan 1. **b.** (Top) Positively defined targets within the rDLPFC using pre-TMS scan 2 (n=44) collected after engaging the threat circuit behaviorally with threat processing, contextual inhibition, and fear conditioning tasks. Targets are mapped to a common MNI template. (Bottom) Density plot showing the distribution of the x, y, and z MNI coordinates of pre-TMS positively defined targets using scan 2 collected following fMRI task.

Figure 4 shows the positive rs-FC guided targets defined after engaging the threat neurocircuitry with a behavioral task. Target topography did not significantly change (F_(1,43)_=0.17, p=0.686, ηp^2^=0.004), suggesting stability in the context of state-dependent dynamics. The average vector of change was 2.9cm (SD 2.1, range 0.14–7.94).

### 3.4 Effect of active versus sham TMS

**Figure 5.**
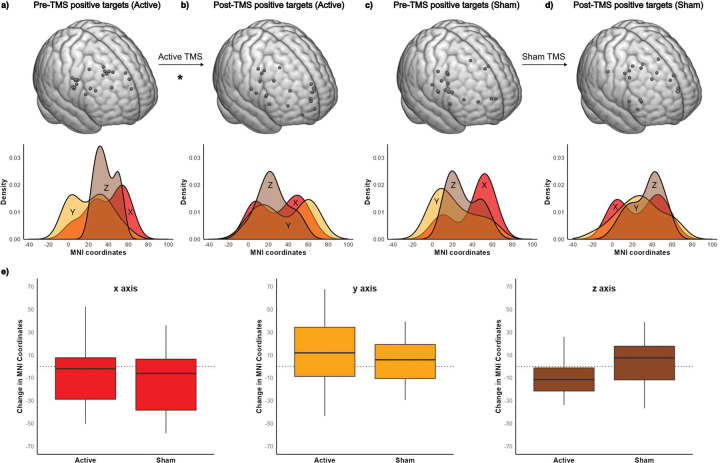
Effect of active *versus* sham TMS on personalized fMRI-guided rDLPFC target **a.** (Top) Targets in the right dorsolateral prefrontal cortex (rDLPFC) with positive correlation in functional connectivity to the right amygdala for pre-TMS scan 1(positively defined targets) in the active TMS group (n=22) mapped to a common MNI template. (Bottom) Density plot showing the distribution of the x, y, and z MNI coordinates of pre-TMS positively defined targets. **b.** (Top) Positively defined targets within the rDLPFC using post-TMS scan in the active group (n=22) mapped to a common MNI template. (Bottom) Density plot showing the distribution of the x, y, and z MNI coordinates of post-TMS positively defined targets. There is a significant change in TMS target topography in the active group from pre- to post-TMS. **c.** (Top) Positively defined targets within the rDLPFC using pre-TMS scan in the sham group (n = 19) mapped to a common MNI template. (Bottom) Density plot showing the distribution of the x, y, and z MNI coordinates of pre-TMS positively defined targets. **d.** (Top) Positively defined targets within the rDLPFC using post-TMS scan in the sham group (n=19) mapped to a common MNI template. (Bottom) Density plot showing the distribution of the x, y, and z MNI coordinates of post-TMS positively defined targets. There is no significant change in TMS target topography in the sham group from pre- to post-TMS. **e.** Box plots show the change from pre-TMS to post-TMS for x (active, mean 2.2cm; sham, mean 2.4cm), y (active, mean 2.7cm; sham, mean 2.0cm) and z (active, mean 1.8cm; sham, mean 1.9cm).

The effect of TMS delivery on topographic location was assessed by comparing the pre-TMS and post-TMS target locations in the active TMS group (n=22) who received twenty 1Hz TMS sessions (36,000 pulses) and the sham TMS group (n=19) in a randomized double blind clinical trial. Figure 5 shows the pre-TMS and post-TMS targets for both the active and sham TMS groups. There was a significantly different effect of sham or active TMS on change in topographic location over time (interaction, F_(2,38)_=3.56, p=0.038, ηp^2^=0.158).

In the sham group (Figure 5A,B), target topography did not show significant changes over time (F_(1,18)_=1.11, p=0.305, ηp^2^=0.058), although individual variability reflected by x, y, and z coordinate change scores was observed with a mean vector change of 4.1 cm (SD=2.0, range 1.25–8.58).

The active TMS group (Figure 5C,D) showed a significant change in TMS topography from pre-TMS to post-TMS (F_(1,21)_=4.60, p=0.044, ηp^2^=0.180) with a mean vector change of 4.3 cm (SD=2.6, range 0.34–8.59).

### 3.5 Exploratory correlations with PTSD symptoms

Exploratory correlation analyses were conducted to test whether change in TMS topography was correlated with clinical change in PTSD. Greater improvement in PTSD symptoms was associated with greater change in the X direction (more medial), r=0.46, p=0.033, and Y direction (more anterior), r=−0.43, p=0.046, but not Z. Additionally, baseline associations were examined. There was no significant correlation between baseline TMS target location (used in the clinical trial) and symptom severity at pre-TMS, post-TMS or symptom change as tested by correlating the x, y, and z MNI coordinates with PCL-5 scores baseline, after TMS, and the change over time (all p’s>0.05).

## 4. Discussion

In this paper, we present our neurocircuitry-based TMS approach for a rs-fMRI-guided individualized target to treat PTSD and other anxiety and negative valence disorders of the amygdala and its circuitry. We addressed unanswered questions regarding its variability and stability, which are important to consider for the use of a patient-specific definition of DLPFC targets using rs-fMRI for precision neuromodulation.

First, the significant variability in target locations between participants supports the topographic heterogeneity of these functional circuits across individuals and justifies the use of patient-specific targeting strategies such as rs-FC-based individualized targeting for TMS.

Second, we demonstrated significant variability between the positive rs-FC versus a negative rs-FC approach, highlighting the importance of considering the neurobiological and technical implications of these different approaches. Furthermore, our data supports the need for empirical validation of both the biological and clinical effects across targeting methods to elucidate what circuit dynamics follow stimulation of one or other targets, and how clinical efficacy and safety may differ.

Third, we provided evidence that the target location did not significantly change after engaging the threat neurocircuitry with relevant fMRI tasks, providing important data for the stability of this TMS targeting strategy in the context of ubiquitous state-dependent dynamics.

Finally, we re-tested the TMS target location after a clinical course of TMS and analyzed the effect of active versus sham TMS in a randomized clinical trial. Therapeutic TMS changes the topography in patients with PTSD symptoms, whereas there was no significant change following sham. Moreover, a greater change in TMS topography in the medial/anterior direction was related to a greater clinical improvement of PTSD symptoms. This research is a significant advancement in characterizing the use of rs-fMRI to identify individualized cortical targets for TMS treatments of amygdala-centric disorders, such as for patients with PTSD symptoms, and justifying (technically and biologically) its use and further exploration.

### 4.1 Variability between subjects

In this study, we defined the TMS target by identifying the coordinates within a largely defined rDLPFC that included Brodmann areas 8, 9, 10, and 46 to allow for individual variability, in contrast to other studies that use a smaller area [e.g., (7,8)]. The value of identifying an ideal individualized area for stimulation with TMS within a small ROI has recently been questioned (28), given the fact that TMS provides stimulation over a wide area. Our targeting data shows significant interpersonal variability, suggesting there is relevance in using a personalized approach to defining the target. Retrospective studies have shown both clinical superiority for individualized targeting (29) and no clinical association between distance to FC group-level target and clinical improvement (30). Studies directly comparing individualized versus standard targets are needed and underway.

### 4.2 Variability between methods

The positive versus negative correlations with rs-FC-guided targeting of the amygdala resulted in significantly different locations, underscoring the importance and implications of analytical choices. Using negative correlation, the targets were identified to be more anterior in the medial PFC compared to the positive correlations with the amygdala. The ventro)medial (vm)PFC is a key region of the threat neurocircuitry and functions to regulate the amygdala and studies suggesting it as an alternative target for PTSD are discussed in ***Supplemental Materials S5.***

### 4.3 Stability after engaging circuit behaviorally

A critical question to determine the clinical use of fMRI-guided targeting is the stability of the target. Functional MRI leads to a variable and generally noisy signal, given the dynamic nature of the biology it measures, and the technical properties of the MRI sequences used to measure the BOLD signal. This has led to questions about the use of single-subject fMRI strategies to define stable targets for TMS. Our data shows target stability after exposing the circuit to state-dependent changes by engaging the circuitry of interest using task paradigms on social threat, contextual inhibition, and fear conditioning. Our data, therefore, suggest good reliability of this target across state-dependent dynamics.

Target stability was previously demonstrated by several groups using multiple MRI scans collected for healthy adults over time as part of the Human Connectome Project (31). Personalized DLPFC targets for different scans per person were defined using rs-FC with the subgenual cingulate cortex. Ning et al. (2019) compared four 15-minute scans on two different days and showed less variability for the inter-day compared to the inter-scan distance (32). Cash et al. (2021) demonstrated that personalized targets can reliably be determined and remain stable over time across 1 year(33). In our study, we extend findings for an amygdala-centric approach. Moreover, our work does not just assess the within-subject variability of cortical targets between two identical scans but explicitly tests the impact of state-dependent dynamics that directly activate the circuitry of interest and are regularly at play in real-world conditions in the patient populations of interest.

### 4.4 Effect of active versus sham TMS

Twenty sessions of active 1Hz TMS to the positively correlated rs-fMRI-defined target changed the topography, a change not observed in the sham codition. It is important to consider the potential implications of the shift in target location following modulation of the targeted circuit with active TMS. First, an additional mid-treatment scan may be needed to update the target, especially if the change in target location may compromise the therapeutic efficacy of TMS, and future studies including a mid-study MRI are needed. Second, it is possible that TMS changes target location as part of the clinical response following modulation of the circuit. In our exploratory analysis, we provide support for this clinical implication by showing a positive correlation between change in target location and clinical improvement. A greater change in TMS target location in the medial anterior direction toward the vmPFC correlated with greater PTSD symptom reduction, possibly suggesting a shift towards more medial prefrontal control over the amygdala. It can be hypothesized that if medial PFC regions gain control over amygdala regulation this could promote effective fear inhibition as a mechanism of recovery(34). Our target location reflects the peak in rs-FC, and modulating the circuit may induce changes in functional architecture. The therapeutic value of change is further supported by other studies that show the clinical effect of TMS is mediated by change in FC (e.g., (35)). Our study focused on target location from pre- to post-TMS based on peak rs-FC in an area that could be reached with TMS. To understand the full impact of TMS on FC during rest and activation of the threat neurocircuitry in from pre- to post-TMS in PTSD, more detailed FC analyses are planned.

In the sham group, we did not observe a significant change over time. However, visualization of the individual-level changes (Figure 5e) shows variability for some participants from the first to the second scan. This variability is expected for several reasons. We used a broad search area, allowing for variability. At times, different targets were identified that all met the criteria and could be considered equally. Additionally, we did not optimize our method for stability but instead optimized for functional feasibility. Others, for example, Ning et al. have shown reliability and stability of targets increased when using a smoothing kernel with a 12mm full-width half maximum. They were able to quantify the topographic variability within and across individuals, demonstrated the benefit of using more data (at least 30 min using Human Connectome Project sequences), and provided suggestions for analytical strategies to minimize within-subject variability (e.g., Gaussian smoothing of final connectivity maps) (32). Other groups have demonstrated the benefits of using more reliable fMRI sequences, such as multi-echo fMRI, in addition to greater data quantity.(36) Further optimization of the pipeline may be possible, but precision and reliability benefits should be considered against feasibility and timing, which was a high priority for us with clinical feasibility in mind. It is therefore notable that with a single echo and straightforward pipeline, we show group-level stability of the TMS target for the specific method we used (i.e., positively correlated peak in the rDLPFC to the right amygdala), and it is not clear if a more elaborate processing pipeline would yield more stable results. It is possible that some of these dynamics are not only method-specific, but also circuit- or node-specific.

### 4.5 Limitations and future directions

First, the data supports amygdala-guided targeting, but different brain regions may map to the cortex with more or less variability and future research is needed to determine stability and variability for other targets. Second, while being comparable to or exceeding other neuroimaging TMS studies, the sample size is modest, and replication in a larger sample with additional timepoints is needed to further validate the results. Third, the approach prioritized feasibility and clinical utility over optimizing the resting state scan analysis, thereby leaving room for further improving precision in targeting. Finally, the scanner upgrade is a methodological limitation, but the finding was replicated across both scanners, providing support for the generalizability of our approach. Finally, while imaging-guided TMS remains debated due to cost and accessibility challenges, it may improve outcomes and efficiency compared to standard approaches. Several trials or naturalistic studies investigating precision TMS for major depression are underway or have recently been completed and show clinical superiority of imaging-guided TMS over scalp-based TMS (22,23). Future research for amygdala-centered TMS is needed.

### 4.6 Conclusion

This study on fMRI-guided TMS supports the potential of personalized circuit-based TMS protocols. Target variability and stability were demonstrated for amygdala rs-FC-guided TMS, and while in this study patients with PTSD symptoms were included, this approach has great potential for other amygdala-centric conditions, such as different anxiety disorders. Unique aspects of the study include the prospective assessment of fMRI-guided targets using a clinical trial with active versus sham TMS and pre- and post-TMS assessments of TMS target locations. The TMS target remains stable despite behavioral engagement of the threat neurocircuit, but a clinical protocol of TMS induced changes that correlated with PTSD symptom improvements. Together, these components provide strong support for the potential of fMRI-guided targeting in TMS.

## Supplementary Material

Supplementary Files

This is a list of supplementary files associated with this preprint. Click to download.

• BPSvanRooijSupplementalMaterials.docx

## Figures and Tables

**Table 1.** Demographic and clinical characteristics

	Sham (n=24)		Active (n=26)		Overall (n=50)	

**Age (in years)**						
Mean (SD)	45.3	(10.1)	38.3	(11.7)	41.7	(11.4)
Median [Min, Max]	45.0	[26.0, 64.0]	36.5	[20.0, 63.0]	42.0	[20.0, 64.0]
**Sex**						
Male	3	(12.5%)	4	(15.4%)	7	(14.0%)
Female	21	(87.5%)	22	(84.6%)	43	(86.0%)
**Gender**						
Man	2	(8.3%)	4	(15.4%)	6	(12.0%)
Woman	20	(83.3%)	21	(80.8%)	41	(82.0%)
Non-binary/Gender-queer	2	(8.3%)	1	(3.8%)	3	(6.0%)
**Race**						
White	15	(62.5%)	15	(57.7%)	30	(60.0%)
Black	6	(25.0%)	9	(34.6%)	15	(30.0%)
Asian	1	(4.2%)	1	(3.8%)	2	(4.0%)
Other/Mixed	2	(8.3%)	0	(0.0%)	2	(4.0%)
Missing	0	(0.0%)	1	(3.8%)	1	(2.0%)
**Ethnicity**						
Hispanic	2	(8.3%)	2	(7.7%)	4	(8.0%)
Non-Hispanic	21	(87.5%)	23	(88.5%)	44	(88.0%)
Missing	1	(4.2%)	1	(3.8%)	2	(4.0%)
**Psychotropic medication**						
Any type	13	(54%)	16	(62%)	29	(58%)
Anti-depressant	12	(20%)	10	(38%)	22	(44%)
Antipsychotic	3	(13%)	2	(8%)	5	(10%)
Stimulant	2	(8%)	6	(23%)	8	(16%)
Anti-anxiety	5	(21%)	5	(19%)	10	(20%)
Mood stabilizer	2	(8%)	5	(19%)	7	(14%)

Biological sex (at birth) and gender identity were ascertained using self-report.

**Table 2.** Scan parameters

Scan	Siemens 3T Trio (n=14)	Siemens 3T Prisma (n=32)

**T1w structural MRI**	TR=2400ms; TE=2.07ms; FA=8°; voxel size=0.8mmx0.8mmx0.8mm	TR=2400ms; TE=2.07ms; FA=8°; voxel size=0.8mmx0.8mmx0.8mm
T2* functional MRI	Number of slices 42; TR=1450ms; TEs=15.0/30.93/46.86/62.79/78.72; voxel size=3.0mmx3.0mmx3.0mm; flip angle= 75deg; multiband acceleration factor=3	Number of slices 39; TR=1450ms; TEs=15.0/31.28/47.54/63.82/80.08; voxel size=3.0mmx3.0mmx3.0mm; flip angle= 75deg; multiband acceleration factor=3
